# The Impact of Feeding Organic Trace Minerals Using Advanced Chelate Technology on Reproductive Efficiency and Blood Parameters in Ghezel Ewes

**DOI:** 10.1002/vms3.70763

**Published:** 2026-01-30

**Authors:** Sedigheh Vatankhah, Marziyeh Ebrahimi, Gholamali Moghaddam, Davoud Kianifard, Akbar Taghizadeh, Reza Asadpour, Mohammad Hassan Nazaran

**Affiliations:** ^1^ Department of Animal Science Faculty of Agriculture University of Tabriz, Tabriz East Azarbaijan Iran; ^2^ Department of Basic Sciences Faculty of Veterinary Medicine University of Tabriz, Tabriz East Azarbaijan Iran; ^3^ Department of Clinical Sciences Faculty of Veterinary Medicine University of Tabriz, Tabriz East Azarbaijan Iran; ^4^ Department of Research and Development Sodour Ahrar Shargh Company Tehran Iran

**Keywords:** ewe, organic trace minerals, reproductive performance

## Abstract

**Background:**

The bioavailability of trace minerals (TM) in a diet is important for improving reproductive performance and fertility parameters.

**Objective:**

This study evaluated the effects of replacing inorganic trace minerals (ITM) with organic trace minerals (OTM), formulated using advanced chelate technology, on the reproductive performance and blood attributes of ewes.

**Methods:**

Forty ewes were subjected to a five‐week intervention in four dietary groups (*n* = 10): (1) OTM: flushed with organic trace minerals (Bonzaplex‐7); (2) ITM: flushed with inorganic trace minerals; (3) NTM: flushed without adding trace minerals; and (4) CON: grazed only on pasture. Oestrus synchronization was performed based on two doses of 75 µg cloprostenol with 12 days interval + 400 IU eCG injection. The number of follicles and pregnancy were determined by ultrasound. Blood samples were collected at predetermined time points during the experimental period (Days 0, 11, 14 and 34).

**Results:**

OTM (80%) and ITM (90%) groups exhibited higher first‐cycle pregnancy rates, as well as improved lambing and twinning rates. OTM demonstrated the greatest efficiency across all reproductive indices. However, follicle number was not affected by the treatments. Also, T3 and T4, as well as triglycerides, were higher in the OTM group as compared with other groups (*p *< 0.05). Serum estradiol, progesterone and antioxidant enzymes were not affected by treatments (*p* > 0.05).

**Conclusions:**

Overall, supplementing advanced chelate technology‐based OTM in the flushing diet improved the reproductive performance of ewes.

## Graphical abstract

Supplementing the flushing diet with organic trace minerals based on advanced chelate technology enhanced the reproductive performance of ewes as compared with inorganic trace minerals.

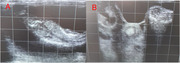

## 1 Introduction

An optimal supply of trace minerals (TM) is essential for the health, production and reproduction of ruminants (Overton and Yasui 2014; Suttle 2022). Additionally, trace element deficiencies have been reported to negatively affect the reproductive efficiency of sheep and goats (Vázquez‐Armijo et al. 2011). Feeding poor or imbalanced TMs may also disturb the antioxidant capacity (Andrieu 2008). Additionally, TMs act as cofactors for enzymes and participate in hormone synthesis, which affects reproduction (Andrieu 2008; Yasothai 2014). The chemical form of TM is a crucial factor that affects its absorption and use in metabolic pathways (Kinal et al. 2005). Studies have shown that the bioavailability of organic trace minerals (OTM) is higher than that of inorganic sources, which allows more mineral absorption (Kinal et al. 2005; Xie et al. 2014; Salami et al. 2016; Shi et al. 2018; Alimohamady et al. 2019; Yaqoob et al. 2020; Farrag et al. 2021). Compared to ITM, supplementing the diet with OTM increased pregnancy rates, improved embryo survival and enhanced prolificacy in ewes, goats and dairy cattle (Uchida et al. 2001; Díaz‐García 2019; Safdar et al. 2020; Farrag et al. 2021; Perry et al. 2021). Additionally, studies have shown that feeding OTM to sheep leads to a higher antioxidant status and improved growth performance compared to ITM (Roy and Samanta 2006; Mousaie et al. 2014; Sethy et al. 2018; Seifalinasab et al. 2022). Chelated minerals, produced based on advanced chelate compound technology, are OTMs bonded to specific organic acids under controlled conditions based on their affinity. These stable, neutral complexes are preserved during the chemical reactions of digestion (Ghasemi et al. 2020; Byrne et al. 2021). Despite the extensive research conducted on OTM, advanced chelate technology has not been employed in the flushing diet of Ghezel ewes. This study aims to address this significant gap in knowledge. A paucity of reports exists concerning the impact of replacing ITM with homologous OTM produced by advanced chelate technology in ewes' flushing diets on reproduction and health. Therefore, the present study was designed to evaluate the effects of OTM produced by advanced chelate technology on ewes' reproductive indicators, hormonal profiles, blood metabolites, antioxidant statuses and enzyme levels.

## 2 Materials and Methods

### 2.1 Experimental Animals and Design

The experiment was done at the Khalat‐Pushan Agricultural Research Station, University of Tabriz, Iran (coordinates: 38°01′54″N 46°23′41″E) from July 2021 to April 2022. Animals (four rams and 40 ewes from the Ghezel breed) were grazed on pasture for 10 h daily and fed a flushing diet (based on the treatments) at 8 PM, based on group housing (10 animals in each cage). During the breeding season, which extends from early August to December, 40 Ghezel ewes with an average body weight of 49.04 ± 7.5 kg, parity ranging from 1 to 4 and BCS between 2 and 3.5 were initially stratified to ensure equal distribution based on body weight and parity. So, BCS was conducted using a standardized 5‐point scale (1 = *emaciated*, 5 = *obese*) with 0.5‐point increments. Also, the live body weights of the ewes were measured using a certified digital scale intended for livestock. Then, they were randomly assigned to one of four dietary treatment groups (10 animals in each group) for a 5‐week experimental period. (1) OTM—grazed on pasture and received a flushing diet supplemented with OTMs (Bonzaplex‐7); (2) ITM—grazed on pasture and received a flushing diet supplemented with inorganic TMs; (3) NTM—grazed on pasture and received a flushing diet without adding TMs (positive control); and (4) CON—grazed on pasture only, without flushing or TM supplementation (negative control). The amount of the flushing diet (300 g) was chosen based on a study by Soroman‑Christian and Juhianinen (2002) and NRC (2007). The energy and protein levels were set according to the study by Mirzaei‐Alamouti et al. (2018). With a 0.3% mineral supplement in the diet, each ewe received 0.9 g of Benzaplex 7 per day during the flushing period, as recommended by the supplement company, the producer of Benzaplex 7. Ewes had access to clean water ad libitum. Upon returning to their holding station (OTM, ITM and NTM), each group received its assigned flushing (group‐wise: 10 animals in each group) diet at 8 PM. The chemical composition and nutritional values of the flushing diet (NRC 2007) are presented in Table 1.

**TABLE 1:** Flushing diet ingredients of Ghezel ewes and rams.

Ingredients of flushing	Percentage
Soybean meal	17.52
Barley grain	68.36
Wheat bran	9.44
Salt	0.50
Calcium carbonate	1.19
Dicalcium phosphate	1.19
Sodium bicarbonate	0.50
Vitamin supplement^a^	1.00
Mineral supplement or filler^b^	0.3
Chemical composition of the flushing diet	
ME (Mcal/kg)	2.4
CP (%)	16
Ca (%)	0.8
P (%)	0.38

^a^ Vitamin supplement composition per kg: 500,000 IU/kg vitamin A, 100,000 IU/kg vitamin D3, 100 mg/kg vitamin E and 400 mg/kg Antioxidant.

^b^ Each kg of mineral supplement in OTM (Bonzaplex‐7) contained 8000 mg/kg Fe; 51,000 mg/kg Zn; 18,000 mg/kg Cu; 28,000 mg/kg Mn; 300 mg/kg Se; 1,700 mg/kg Co; 500 mg/kg Cr. Each kg of mineral supplement in ITM treatment had the inorganic form of trace minerals equivalent to the minerals presented in Bonzaplex‐7.

**FIGURE 1:**
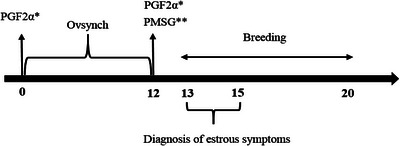
Oestrus synchronization protocol of Ghezel ewes.

All ewes were synchronized using a modified cloprostenol protocol consisting of two intramuscular injections of 75 µg d‐cloprostenol (Royan Drug Pharmacy Institute, Semnan, Iran) administered 12 days apart. The second d‐cloprostenol injection was followed immediately by 400 IU equine chorionic gonadotropin (eCG) (Gonaser; Hipra, Spain) on Day 12 of the experiment (Figure 1). This protocol was selected based on its proven efficacy in small ruminants (Hasani et al. 2018). Rams were introduced to the herd between Days 13 and 20 of the experiment for the first oestrus breeding period. Ewes showing signs of oestrus were introduced to a second ram on Day 17 to maximize conception opportunities.

### 2.2 Blood Sampling and Assay

Blood samples were collected via jugular venipuncture from each animal on Days 0 (start of feeding flushing diet and synchronization), 11 (24 h before injection of the second dose of d‐cloprostenol), 14 (48 h after injection of the second dose of d‐cloprostenol) and 34 (at the end of feeding flushing diet). Blood samples were collected into non‐heparinized tubes, allowed to clot for 2 h at room temperature and centrifuged (3000 × *g* for 20 min) to separate serum. Samples were stored at −20°C until hormones, metabolites, enzymes and antioxidant analysis. In addition, whole blood samples were collected by jugular venipuncture into sterile EDTA tubes on day 34 of the experiment and stored at −20°C to evaluate blood superoxide dismutase (SOD), glutathione peroxidase (GPx) and haemoglobin (Hb) concentrations.

Serum glucose, total protein, cholesterol and triglyceride, as well as serum alkaline phosphatase (ALP) and cholinesterase, were evaluated by the Enzymatic, Colourimetric method and spectrophotometrically by using reagents from Sigma Diagnostics (Pars Azmon Co, Tehran, Iran).

Hormone kits of oestrogen (Product No. 4925‐300A, Monobind, USA), progesterone (Product No. 4825‐300A, Monobind, USA), T3 (Product No. 14003, Pishtaz TEB Diagnostics, Iran) and T4 (Product No. 14003, Pishtaz TEB Diagnostics, Iran) were used to evaluate respected hormones based on the enzyme‐linked immunosorbent assay (ELISA) method by ELISA reader (Hiperion Microplate Reader, Germany). Intra‐assay coefficients of variation for progesterone, oestrogen and insulin were 0.42%, 0.13% and 0.21%, respectively. Also, intra‐assay coefficients of variation for serum T3 and T4 were 0.06% and 0.47%, respectively.

Hb concentration was measured using the cyanmethaemoglobin method (Cannan 1958).

Malondialdehyde (MDA) concentration was measured by the thiobarbituric‐acid reaction (ZellBio, Germany) based on a colourimetric assay (Esterbauer and Cheeseman 1990).

GPx activity, SOD activity and total antioxidant capacity (TAC) were measured by a respective commercial kit (Randox, Crumlin, UK). The absorbance was recorded by a spectrophotometer (T80 UV/VIS PG Instruments Ltd, UK) based on the kit‐suggested wavelengths.

### 2.3 Ultrasound Examination

**FIGURE 2:**
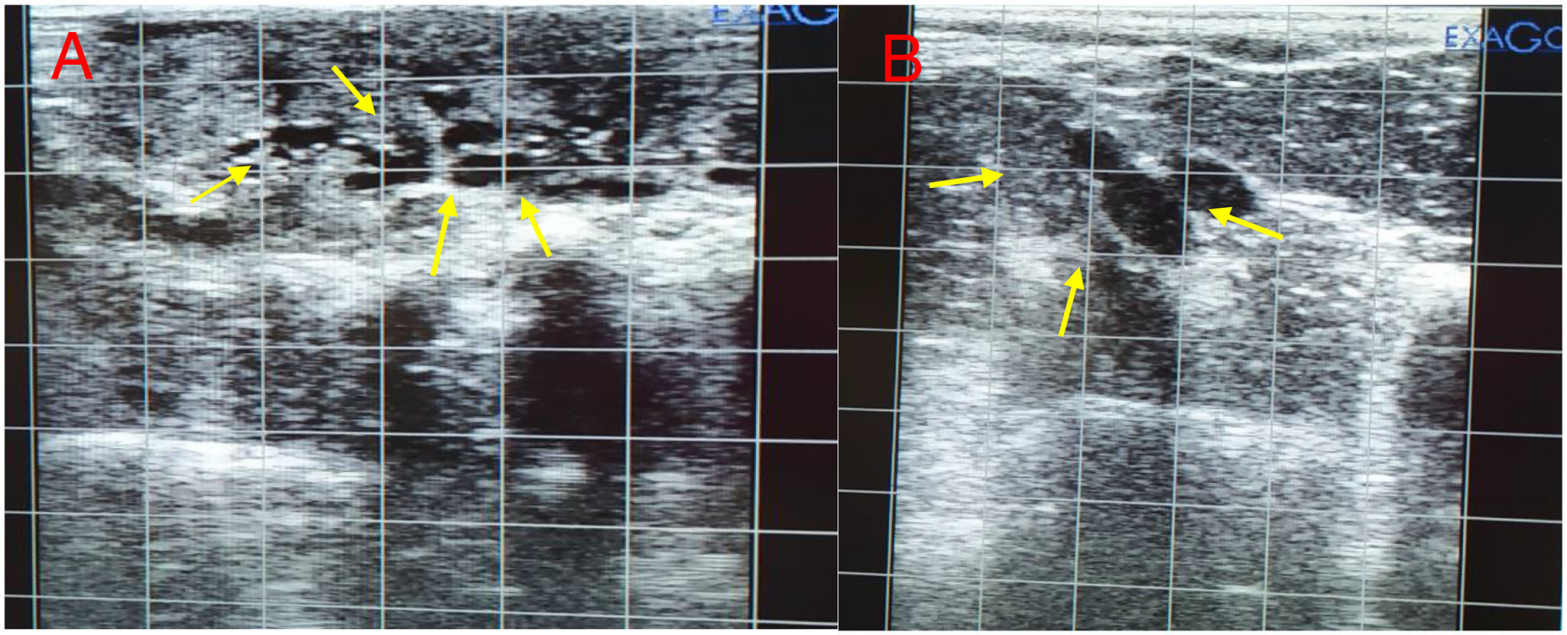
Ultrasound images on Day 15 of the experiment (72 h after synchronization) show the number of follicles in Ghezel ewes. (A) Yellow arrows show the developing follicles. (B) Yellow arrows show the preovulatory follicles.

Real‐time B‐mode ultrasonography (EXAGO, ECM Company, NANUK 930, French) equipped with a 7.5 MHz linear array transducer was used to diagnose the number of follicles on the right ovary of ewes on Day 15 of the experiment (72 h after synchronization), (Figure 2). Briefly, the linear transducer was inserted into the rectum after removing faecal pellets. Ewes underwent ultrasound examination while restrained in a holding crate in a standing position. The rectal probe was inserted into the rectum, and the ovaries were viewed.

**FIGURE 3:**
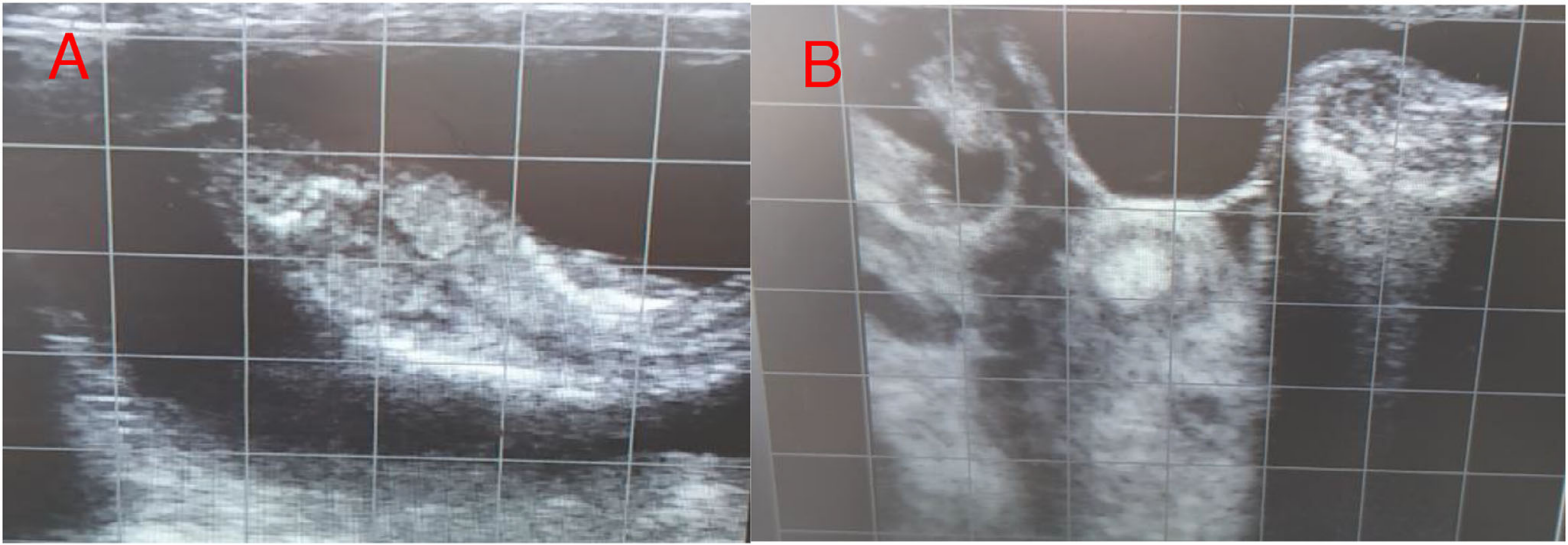
Ghezel ewe's ultrasound: first pregnancy examination on Day 40 after ram introduction (first oestrus), (B) the second oestrus pregnancy examination was done on Day 70 after ram introduction (second oestrus). Ultrasound of a developing ovine foetus showing ribcage, head, uterus, tissue and amnion. Obtained using a transrectal 7.5 MHz linear array probe. It is displayed using a real‐time B‐mode ultrasound scanner.

Pregnancy was also examined twice. The first pregnancy examination during the oestrous cycle was conducted via transrectal ultrasonography on Day 40 after ram introduction. The second examination was conducted on Day 70 after ram introduction. Note that only non‐pregnant ewes from the initial examination were evaluated for pregnancy in the second ultrasound (Figure 3).

### 2.4 Determination of Lambs’ Weight and Reproductive Performance

After parturition, lamb numbers and their weight were recorded. Then, all reproductive parameters were calculated based on the formula presented by Hasani et al. (2018).

### 2.5 Statistical Analysis

Reproductive performance data were analysed using the LOGISTIC procedure of SAS software (SAS Institute 2008). Blood factors (except for oestrogen and progesterone), BCS, number of follicles, and lambs’ weight were analysed by GLM procedure (Model 1) of SAS software, and Duncan's multiple range test was used for comparing treatments. *p* < 0.05 was considered statistically significant. Also, a normality test was done for the data. Blood oestrogen and progesterone concentrations at four different times were analysed using the MIXED procedure (Model 2) of SAS software (SAS Institute 2008).

$$\mathrm{Model}1:Y_{ij}=\mu +F_{i}+\alpha \left(C_{ij}-{\bar{C}}_{00}\right)+e_{ij}$$

$$\begin{array}{ccc} & & \mathrm{Model}2:Y_{\mathrm{ijkl}}=\mu +F_{i}+T_{j}+FT_{\mathrm{jj}}\\ & & \;+\mathrm{Anima}l_{k}\left(F_{i}\right)+\alpha \left(C_{ijk}-{\bar{C}}_{000}\right)+e_{\mathrm{ijkl}} \end{array}$$

where *Y* (*Y_ijkl_* or *Y_ij_*) is the dependent variables; *F_i_* is the fixed effect of flushing protocols; *T_j_* is the fixed effect of time of blood sampling; + Animal*_k_* (*F_i_*) is the random effect of animal in flushing protocols; *FT_jj_* is the interaction effect of treatment by time; *α* is the linear regression coefficient of ewes’ blood factors or BCS ($C_{ijk}\;or\;C_{ij}$) from primary blood measurements or BCS (${\bar{C}}_{000}\;or{\bar{C}}_{00}$); and *e* (*e_ijkl_* or *e_ij_*) is the experimental error.

## 3 Results

### 3.1 Reproductive Performance

Reproductive performance parameters during the breeding season are presented in Table 2. The results of the present experiment demonstrated higher pregnancy, lambing and twinning rates in the OTM and ITM groups compared with the other groups, with the OTM group showing superior performance across these reproductive indices. Also, results indicated the lowest abortion rate (0%) in the OTM group as compared with other treatment groups (*p *> 0.05). However, the number of follicles was not significantly affected by the dietary treatments. In addition, serum levels of triiodothyronine (T3), thyroxine (T4) and triglycerides were significantly higher in the OTM group compared with the other groups (*p* < 0.05). No significant differences were observed in serum estradiol, progesterone or antioxidant enzyme levels among the treatment groups.

**TABLE 2:** Effect of organic and inorganic minerals on reproductive parameters of Ghezel ewes during the flushing period.

	Treatments				
Reproductive parameters	OTM (*n* = 10)	ITM (*n* = 10)	NTM (*n* = 10)	CON (*n* = 10)	*p* value
Follicle number (mean ± SE)	3.20 ± 0.23	2.70 ± 0.23	3.50 ± 0.23	3.10 ± 0.23	0.12
Number of pregnant ewes in the first oestrus (head)	8	9	5	4	−
Pregnancy rate in the first oestrus (%)	80	90	50	40	0.09
Total number of pregnant ewes (head)	10	10	7	10	−
Total pregnancy rate (%)	100	100	70	100	0.9
Number of abortion (head)	0	3	2	4	
Abortion rate (%)	00	30	28.57	40	0.8
Number of lambs in the first oestrus (head)	13	10	6	4	−
Lambing rate in the first oestrus (%)	130 ^a^	100 ^ab^	60 ^ab^	40 ^b^	0.03
Total number of lambs (head)	16	11	7	9	−
Total lambing rate (%)	160 ^a^	110 ^ab^	70 ^b^	90 ^b^	0.04
Twinning rate in the first oestrus (%)	76.92	80	33.33	00	−
Total twinning rate (%)	75	72.72	28.57	44.44	−
Prolificacy in the first oestrus (%)	162.5	166.67	120	100	−
Total prolificacy (%)	160	157.14	116.67	128.57	−

*Note*: Pregnancy rate (number of pregnant ewes/total number of ewes × 100); Abortion rate (number of ewes that were pregnant by ultrasonography and did not lamb/total number of ewes that were pregnant × 100); lambing rate (number of lambs/ewes joined × 100); twinning rate (lambs born as twins or triplets/ whole lambs× 100); prolificacy (number of lambs/ewes lambing × 100).

Abbreviations: CON, control group; ITM, flushing with inorganic trace minerals; NTM, flushing without TM; OTM, flushing with organic trace minerals.

### 3.2 Lambs̕ Birth Weight and Ewes̕ Body Condition Score

The effects of the dietary treatments on lamb birth weight and ewe body condition score are presented in Table 3. The treatments significantly influenced the birth weight of lambs conceived during the first oestrus cycle (*p* < 0.01), with lower birth weights observed in the OTM and ITM groups compared with the NTM and CON groups. However, neither the total lamb birth weight (including lambs born from ewes fertilized in both the first and second oestrus cycles) nor the ewes’ body condition scores were significantly affected by the flushing treatments.

**TABLE 3:** Effect of organic and inorganic minerals on birth weight and ewes’ body condition score of Ghezel ewes (mean ± SE).

Traits	Treatments				*p* value
	OTM (*n* = 10)	ITM (*n* = 10)	NTM (*n* = 10)	CON (*n* = 10)	
Lamb birth weight in the first oestrus (kg)	3.68 ± 0.17^b^	3.99 ± 0.19^b^	4.75 ± 0.25^a^	4.91 ± 0.31^a^	< 0.01
Total lamb birth weight (kg)	3.96 ± 0.18	4.12 ± 0.22	4.71 ± 0.27	4.68 ± 0.24	0.06
BCS of ewes	3.14 ± 0.17	3.35 ± 0.17	3.24 ± 0.18	2.70 ± 0.17	0.06

*Note*: Means within the same row with different letters differ significantly (*p *< 0.05).

Abbreviations: BCS, body condition score; CON, control group; ITM, flushing with inorganic trace minerals; NTM, flushing without TM; OTM, flushing with organic trace minerals.

### 3.3 Blood Parameters

The results related to all blood parameters are shown in Table 4. Based on the results, the flushing treatments did not affect the plasma concentrations of total protein, glucose and cholesterol (*p* > 0.05) (Table 4). However, serum triglyceride concentration was significantly affected by treatments (*p* < 0.05), with the highest amount of triglyceride observed in the OTM and CON groups, while the lowest was observed in the ITM group. In addition, T3 concentration was significantly affected by the treatments, and the highest concentration was observed in the OTM, NTM and CON groups (*p* < 0.05), with no significant difference among the three groups (Table 4). Serum concentration of T4 was also affected by flushing treatments (*p* < 0.01), and the highest amount was observed in OTM and ITM treatment groups (Table 4).

**TABLE 4:** Effect of organic and inorganic minerals on blood parameters of Ghezel ewes during the flushing period (mean ± SE).

	Treatments				
Parameters	OTM (*n* = 10)	ITM (*n* = 10)	NTM (*n* = 10)	CON (*n* = 10)	*p* value
TP (g/dL)	7.52 ± 0.17	7.03 ± 0.17	7.53 ± 0.17	7.40 ± 0.17	0.14
Glucose (mg/dL)	44.30 ± 3.6	40.10 ± 3.6	38.50 ± 3.6	35.70 ± 3.6	0.40
TG (mg/dL)	44.0 ± 2.6^a^	31.50 ± 2.6^c^	34.70 ± 2.6^cb^	40.40 ± 2.6^ab^	0.01
Cholesterol (mg/dL)	60.90 ± 3.74	59.30 ± 3.74	58.10 ± 3.74	64.10 ± 3.74	0.70
T_3_ (ng/dL)	2.14 ± 0.13^a^	1.57 ± 0.13^b^	1.91 ± 0.14^ab^	1.83 ± 0.13^ab^	0.03
T_4_ (ng/dL)	6.38 ± 0.26^a^	6.02 ± 0.26^a^	0.83 ± 0.28^b^	0.91 ± 0.26^b^	< 0.01
Estradiol (ng/mL)	44.24 ± 9.59	58.77 ± 10.04	48.32 ± 10.04	82.05 ± 10.19	0.07
Progesterone (ng/mL)	4.75 ± 0.78	4.50 ± 0.76	4.35 ± 0.75	3.81 ± 0.74	0.84
ALP (U/L)	100 ± 28.10	175.10 ± 25.13	146.60 ± 25.13	145.50 ± 25.13	0.28
Cholin‐esterase (U/L)	258.77 ± 22.26	245.11 ± 22.26	242.10 ± 21.12	268.20 ± 21.12	0.81
MDA (nmol/mL)	1.80 ± 0.24	2.54 ± 0.24	1.90 ± 0.24	2.34 ± 0.24	0.09
GPx (IU/gHb)	60.18 ± 4.01	74.16 ± 4.01	71.51 ± 4.01	61.64 ± 4.01	0.06
SOD (U/gHb)	1393.82 ± 82.90^c^	1704.43 ± 82.90^a^	1436.30 ± 82.90^bc^	1673.07 ± 82.90^ab^	0.03
TAC (nmol/L)	0.25 ± 0.02	0.29 ± 0.02	0.31 ± 0.02	0.33 ± 0.02	0.07
Hb (g/dL)	15.66 ± 0.70	13.96 ± 0.70	13.34 ± 0.70	13.26 ± 0.70	0.09

*Note*: Means within the same row with different letters differ significantly (*p *< 0.05).

Abbreviations: ALP, alkaline phosphatase; CON, control group; GPx, glutathione peroxidase; Hb, haemoglobin; ITM, flushing with inorganic trace minerals; MDA, malondialdehyde; NTM: flushing without TM; OTM, flushing with organic trace minerals; SOD, superoxide dismutase; T3, triiodothyronine; T4, thyroxine; TAC, total antioxidant capacity; TG, triglyceride; TP, total protein.

**FIGURE 4:**
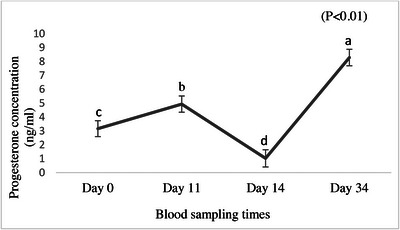
The effect of different blood sampling times on serum progesterone concentration in Ghezel ewes. Days 0 (Start of feeding flushing diet and synchronization), 11 (24 h before injection of the second dose of d‐cloprostenol), 14 (48 h after injection of the second dose of d‐cloprostenol) and 34 (at the end of feeding flushing diet).

Estradiol concentration was not affected by mineral‐supplemented flushing diets (*p* > 0.05), though tended to be higher in the CON group (Table 4). Progesterone concentration was not affected by flushing treatments (*p* > 0.05) (Table 4); though, time had a significant effect on progesterone concentration (*p* < 0.01; Figure 4).

Also, serum ALP and cholinesterase were not affected by treatments (*p *> 0.05) (Table 4).

Plasma concentration of MDA, as a marker of oxidative stress, was not affected by experimental treatments (*p *> 0.05), but the amount tended to decrease in OTM treatment compared with other groups (Table 4). The effect of treatments on the activities of GPx and the value of TAC were not significant (*p *> 0.05), though the SOD activity was markedly lower in the OTM compared with other treatments (*p *< 0.05) (Table 4). Although treatments did not significantly affect Hb concentration (*p *> 0.05), the Hb level of ewes tended to be higher in the OTM group as compared with other groups.

## 4 Discussion

### 4.1 Reproductive Performance

We hypothesized that OTM feeding would increase the number of developing ovarian follicles, but the data from the present experiment showed that there was no difference in the number of follicles between the treatments and the control group. Similar to our results, Lamb et al. (2008) showed no effect of supplementing organic and inorganic minerals on the number of follicles, follicle size and number of ovulated follicles. Conversely, our results were not consistent with the results of the other studies using inorganic or OTMs (Monem and El‐Shahat 2011; Khanthusaeng et al. 2022).

Pregnancy rate in the first oestrus tended to be higher in OTM (80%) and ITM groups (90%) as compared with NTM (50%) and CON (40%) groups. However, the total pregnancy rate was not affected by treatments. Furthermore, the lowest abortion rate (0%) was observed in the OTM group. Resembling the present study, Díaz‐García (2019) reported that selenium supplementation of ewes’ diet (during flushing and gestation period) from either organic or inorganic sources had no effect on pregnancy rate. In another study, dietary selenium supplementation in ewes insignificantly increased pregnancy rate (Sánchez et al. 2008). Also, Thomas (2022) reported similar pregnancy rates between feeding inorganic and organic (66.6% and 62.5%, respectively) mineral treatments in cows. Resemble to the present study, Perry et al. (2021) reported that supplementing heifers’ diet with Co, Cu, Mn and Zn from either organic or inorganic sources had no effect on pregnancy rate, but a tendency to reduce embryonic loss was observed in heifers that received organic sources. Also, Rabiee et al. (2010) found that incorporating OTM, specifically copper (Availa‐4 and Plex‐4), into the diets of dairy cows significantly improved their reproductive performance. This dietary change led to a reduction of 13.5 days in the duration cows remained open and decreased the number of services needed per conception by 0.27. Furthermore, it enhanced the pregnancy rate by 7% within the first 150 days of lactation (Rabiee et al. 2010). The present study revealed that the organic mineral source had no impact on pregnancy rates, but it might enhance foetal health as pregnancy advances.

In the current study, the highest lambing rate was observed in the OTM group, while twinning rate and prolificacy were both higher in the OTM and ITM groups as compared with other groups. The essential TMs are known to influence embryonic and foetal survival, thereby influencing the number and size of offspring at parturition (Hostetler et al. 2003). The increase in lambing rate in the OTM group can probably be related to the higher content of available selenium in the ewes provided by organic minerals as compared with inorganic form of minerals, which is in agreement with previous studies (Steen et al. 2008; Díaz‐García 2019). Also, Sánchez et al. (2008) reported higher lambing and twinning rate, in ewes supplemented by 0.5 mg/kg of selenomethionin. Safdar et al. (2020), by supplementing organic selenium in flushing diets of Afshari ewes, reported higher fertility success and lambing rate. Also, Daghigh Kia et al. (2019) demonstrated that Ghezel ewes experienced higher rates of lambing and fewer cases of abortion when organic minerals were used instead of inorganic ones. Díaz‐García (2019) reported that multiparous ewes supplemented with selenium (organic vs. inorganic) were not different in prolificacy rate (lambing rate and twinning rate).

OTMs are more bioavailable than ITMs in ruminants, such as ewes, because they are resistant to rumen degradation (Byrne et al. 2021; Byrne and Murphy 2022), form fewer complexes with dietary antagonists (Byrne et al. 2021; Weiss and Hansen 2024) and are more easily absorbed in the intestines (Vigh et al. 2023). These characteristics result in higher tissue concentrations (Steen et al. 2008; Zhang et al. 2024), lower excretion (Steen et al. 2008) and enhanced physiological effects, particularly on reproduction, under conditions of deficiency or stress (Díaz‐García 2019; Safdar et al. 2020; Perry et al. 2021). During ovulation, manganese (Mn) improves oocyte quality and the rate of ovulation by facilitating the synthesis of cholesterol (Xie et al. 2014), a precursor to steroid hormones such as progesterone (Hostetler et al. 2003) and estradiol, and by supporting the function of the corpus luteum (CL). Zinc (Zn) maintains the integrity of reproductive tissues by supporting nucleic acid metabolism and gonadal cell division (Ertek et al. 2010). Copper (Cu) activates enzymes that are essential for steroidogenesis and stimulates the release of GnRH and FSH (Vázquez‐Armijo et al. 2011; Roychoudhury et al. 2016), which increases estradiol production in granulosa cells (Nikhil Kumar Tej et al. 2021). Also, selenium (Se) provides antioxidant protection to ovarian tissue against oxidative damage through selenoproteins such as GPx (Brown et al. 2000; Christensen et al. 2015; Novoselec et al. 2022). During pregnancy, OTMs mitigate early embryonic mortality and increase pregnancy rates by incorporating selenium (Se) into GPx to reduce reactive oxygen species (ROS), which surge during the initial phases of gestation (Novoselec et al. 2022). This protects the placenta and embryo. Zinc (Zn) bolsters epithelial integrity for implantation and uterine repair (where deficiencies lead to resorption) (Nair et al. 2023). Copper (Cu) fosters connective tissue development and maintains hormonal levels (oestrogens and LH) (Vázquez‐Armijo et al. 2011; Roychoudhury et al. 2016). Manganese (Mn) enables enzymatic frameworks for foetal growth (Byrne and Murphy 2022). Overall, both OTM and ITM forms compensate for element deficiencies. However, the OTM form has more reliable reproductive effects due to its different uptake (binding to carbon carriers) and reduced limiting interactions. Future studies with larger sample sizes are recommended to confirm this difference.

### 4.2 Lambs’ Birth Weight and Ewes’ Body Condition Score

In the current study, lambs born in OTM and ITM groups had lower birth weight as compared with NTM and CON groups. These results are concomitant with higher twining rates in these groups. It was reported that twin foetuses have a slower intrauterine growth rate than singleton foetuses, which is probably due to the lower availability of oxygen and nutrients, because of the lower capacity of the placenta (De Matteo et al. 2008). Then, twins are lighter than singletons at birth (De Matteo et al. 2008).

In the present study, ewes’ BCS were not affected by flushing treatments, though the CON group had the lowest BCS, which could be a result of not receiving the flushing diet. Similar to our results, Amanlou et al. (2020) observed that feeding various sources of TM (Zn, Cu, Mn, Se and Co) from 5 weeks before parturition to 5 weeks after parturition had no effect on the BCS of ewes. Also, it was reported that neither TM supplementation nor sources affected BCS in dairy cows (Yasui et al. 2014).

### 4.3 Blood Parameters

Blood biochemical characteristics, such as glucose, triglyceride, total protein and cholesterol levels, are reliable indicators of animal health (Abdallah et al. 2019). In the current study, the treatments did not affect the plasma concentrations of total protein, glucose and cholesterol. Similar studies on feeding organic zinc sources reported no changes in serum cholesterol, glucose (Feizdar Barabadi et al. 2025), and total protein levels in sheep (Sethy et al. 2018) and Holstein dairy cows (Sobhanirad and Naserian 2012). In another study, Kinal et al. (2005) reported no effect of organic minerals (Cu, Mn, Co and Zn bound with amino acid) on total protein in the serum of cows. Other research by feeding Cu‐Met (Garrine et al. 2019), Se‐Met and/or Cr‐Met (Mousaie et al. 2014), Zn‐Met (Sobhanirad and Naserian 2012), also reported no effect on serum cholesterol level.

Although the ITM group had the lowest triglyceride concentration, no significant differences were observed between the CON and OTM groups. However, previous studies have shown that different forms of organic minerals in sheep can reduce plasma triglyceride concentrations (Uyanik 2001; Yan et al. 2008; Zhou et al. 2022). It has been proposed that chromium may lower triglyceride levels by enhancing the liver's ability to transport them (Yan et al. 2008). Chromium is an integral part of the glucose tolerance factor (GTF) (Yan et al. 2008), which boosts the sensitivity of cells to insulin. It influences lipid metabolism by triggering insulin signalling, controlling the expression of genes related to lipid metabolism and enhancing the function of lipoprotein lipase (LPL) (Seifalinasab et al. 2022; Nair et al. 2023). Additionally, Christensen et al. (2015) demonstrated the impact of selenium on cholesterol and triglycerides. Selenium, by means of GPx, reduces oxidative stress that disrupts insulin signalling and encourages the creation of new lipids (Brown et al. 2000; Christensen et al. 2015; Nair et al. 2023; Zhang et al. 2024). During the reproductive phase of ruminants such as ewes, chelate supplements (OTM) provide controlled mineral and metabolic release, unlike inorganic forms (ITM), which cause sudden changes through rapid ion release (Kinal et al. 2005; Byrne et al. 2021). The balance of selenium and chromium availability in the OTM group appears to have caused a slight increase in triglyceride concentration. In the ITM group, however, these levels of selenium and chromium reduced triglyceride concentration.

Based on the results, the T3 concentration was highest in the OTM group, while the T4 concentration was highest in the OTM and ITM groups. Selenium is present in deiodinase enzymes, and selenium supplementation increases plasma T3 concentrations (Erdoğan et al. 2017; Farrag et al. 2021). Mousaie et al. (2014) found that lambs fed Se‐Met (1.5 mg/kg) and Cr‐Met (0.8 mg/kg) supplements had lower T4 and higher T3 concentrations due to increased T4‐to‐T3 conversion. Similar results were reported by Ebrahimi et al. (2009) in suckling calves fed 0.3 mg/kg of Se from Sel‐Plex. However, several trace elements, such as copper, iron and zinc, are essential for the synthesis and metabolism of thyroid hormones (Nazifi et al. 2010). Thyroid peroxidase is a heme‐dependent protein that catalyses the first two steps of thyroid hormone synthesis. Thus, iron supplementation can improve the activity of this enzyme (Zhou et al. 2022). It has also been stated that the action of hepatic deiodinase decreased in cases of iron deficiency (Zimmermann 2006). Asadi et al. (2022) reported higher T3 and T4 concentrations in suckling lambs when they supplemented their milk with organic iron. A number of investigations have discovered that a reduction in zinc levels is associated with hypothyroidism, while an elevation in zinc levels is linked to hyperthyroidism (Ertek et al. 2010). Additionally, it was suggested that Zn is necessary for the function of the enzyme 5′‐deiodinase (Mion 2022). Alimohamady et al. (2019) reported an increase in serum T3 and T4 concentration and a decrease in the ratios of T3 to T4 due to supplementation of 30 mg Zn/kg diet as organic and/or inorganic in lambs. In the study by Nazifi et al. (2010), a favourable correlation was identified between the concentrations of Zn and Mn and the fT3 level. Based on the current findings, the higher concentrations of T3 and T4 observed in the OTM group may be associated with increased synthesis and secretion of these hormones, which could be attributed to the greater mineral availability in the OTM group compared to the ITM group.

In the present study, the estradiol concentration tended to be higher in the CON group, while the OTM group had the highest non‐significant amount of progesterone. TMs play a vital role in proper follicle growth and maintaining pregnancy by influencing the secretion of reproductive hormones (Vázquez‐Armijo et al. 2011). Specifically, manganese plays a role in cholesterol synthesis, which is necessary for the production of steroids such as progesterone, oestrogen and testosterone (Xie et al. 2014). Zinc is also crucial for the expression of reproductive hormones, a process that involves specific proteins (Alimohamady et al. 2019). Furthermore, copper plays a crucial role in the synthesis and maintenance of proper levels of follicle‐stimulating hormone in the blood (Vázquez‐Armijo et al. 2011). Copper has been shown to stimulate the release of GnRH and FSH, which in turn triggers the synthesis and release of estradiol (Roychoudhury et al. 2016). Moreover, copper may be steroidogenic, promoting estradiol synthesis by increasing the aromatase enzyme, which converts androgens to estradiol in ovarian granulosa cells (Nikhil Kumar Tej et al. 2021). Rutigliano et al. (2008) found that estradiol levels increased with follicle diameter during Ovsynch, but selenium sources (sodium selenite and/or selenized yeast) did not affect estradiol concentrations. However, dietary supplementation with OTMs increased estradiol concentration in serum (Kujur et al. 2016; Shi et al. 2018; Daghigh Kia et al. 2019; Safdar et al. 2020) and follicular fluid (Khanthusaeng et al. 2022) of ruminants. Progesterone, which is secreted by the placenta and CL, is crucial for establishing and maintaining pregnancy (Shi et al. 2018). Subsequent to ovulation, the CL is formed and the concentration of progesterone concomitantly rises (Shi et al. 2018). Manganese plays a critical role in the secretion of progesterone (Hostetler et al. 2003). Previous studies of ewes showed that feeding selenium during the flushing period increased the concentration of progesterone three weeks after mating (Kujur et al. 2016; Daghigh Kia et al. 2019; Safdar et al. 2020). According to the results of the present study, the tendency toward a higher average estradiol concentration in the CON group, accompanied by a lower progesterone concentration, was related to a lower pregnancy rate in this group. Conversely, the OTM group had a non‐significant increase in serum progesterone and a lower estradiol concentration, which was related to a higher pregnancy rate.

In the present research, the concentrations of ALP and cholin‐esterase in ewes were not affected by the treatments. ALPs are cell membrane metalloenzymes, which its activity in serum used as a marker for bone formation to evaluate osteoblastic activity (Turan et al. 2011). A decrease in serum ALP with advancing pregnancy was also reported in ewes (Gürgöze et al. 2009). Similar to the present results, Gholami et al. (2021) reported no significant difference between inorganic TMs and OTMs with control group on the concentration of ALP in the serum of cows. Although several studies reported no significant effect of organic minerals on ALP concentration (Aksu and Ozsoy 2010; Yaqoob et al. 2020; Toghdory et al. 2023; Feizdar Barabadi et al. 2025), other studies reported positive effects of OTMs on serum ALP concentration of animals (Liu et al. 2016; Alimohamady et al. 2019). Cholinesterase is synthesized in the liver and its low levels are caused by several diseases, especially by liver disease and malnutrition (Vorhaus and Kark 1953). This enzyme hydrolyses acetylcholine into choline (and acetic acid), which is the main component of the cholinergic system, and this enzyme widely distributed in the central nervous system and also in red blood cells, platelets and lymphocytes (Çokuğraş 2003). Similar to the present results, it was indicated that blood cholinesterase was not affected by reproductive status of ewes (Antunović et al. 2004).

In the present study, the plasma concentration of malondialdehyde tended to be lower in the OTM treatment group than in the other groups. Additionally, SOD activity in the OTM treatment was reduced relative to that in the other groups, whereas GPx and TAC were not affected by the treatments. The effectiveness of protection against oxidative stress depends on the presence of sufficient amounts of Zn, Cu and Mn (Alimohamady et al. 2019; Yaqoob et al. 2020; Zhang et al. 2024; Feizdar Barabadi et al. 2025). Disturbance in the homeostasis of elements such as Fe, Cr, Cu and Co leads to the production of reactive radicals (superoxide anion and nitric oxide), which leads to a state of oxidative stress (Nair et al. 2023; Zhang et al. 2024; Feizdar Barabadi et al. 2025). It has been shown that depletion of Zn may lead to an increase in the production of free radicals and a decrease in GPx and SOD activities (Nair et al. 2023; Zhang et al. 2024; Feizdar Barabadi et al. 2025). OTM (Se‐Met and Cr‐Met) decreased MDA levels in Baloch lambs at 9 weeks (Mousaie et al. 2014). Also, lower serum concentration of MDA and higher GPx content was observed with feeding Cr‐Met in lambs (Seifalinasab et al. 2022) and Se‐Yeast in sheep (Qiu et al. 2022). Conversely, adding organic (oligosaccharide‐chelated OTM) and inorganic forms of zinc and copper to lamb diets led to an enhancement in SOD and GPx activity (Feizdar Barabadi et al. 2025). According to reports, the levels of organic and inorganic minerals differ between enzymes and tissues (Nair et al. 2023; Zhang et al. 2024). Inorganic minerals, such as sulphates and oxides, release ions quickly, which stimulates antioxidant enzymes. Organic minerals, such as chelates, take longer to break down and have slower effects over short periods (Alimohamady et al. 2019; Safdar et al. 2020; Nair et al. 2023; Zhang et al. 2024). Sodium selenite boosts Se availability for GPx synthesis in the short term, whereas organic Se (e.g., Se‐yeast) is better absorbed and stored, ensuring stable nutrient levels (Novoselec et al. 2022; Nair et al. 2023; Zhang et al. 2024). In addition, the rising demand for TMs (Se, Zn, Cu, Co, Fe, Mn and I) is associated with physiological and environmental stress, as well as reproductive performance and immunity (Suttle 2022). The reduction in the concentration of mineral elements during pregnancy occurs because of the heightened transfer of these elements to the foetus, which utilizes them for the synthesis of antioxidant enzymes (Spencer et al. 2015). Oxidative stress increases in early pregnancy due to the high metabolic rate in the placenta (Al‐Gubory et al. 2010) and the increased production of ROS, which may be linked to embryo development (Myatt 2006). Changes in SOD and GPx activities in the sheep CL during pregnancy (Al‐Gubory et al. 2004) and the oestrous cycle (Al‐Gubory et al. 2005) are likely associated with ROS production in luteal cells. The present research indicated a trend of reduced MDA and a significant reduction of SOD activity in the OTM group, while GPx and TAC remained unchanged. This implies that organic minerals might offer superior defence against oxidative stress due to their enhanced bioavailability and lasting effects, particularly during pregnancies with heightened mineral requirements. Nonetheless, the five‐week treatment period may have been insufficient to fully observe the impact on antioxidant enzyme activity. Additionally, variations in tissue and enzymatic responses to different mineral forms (Alimohamady et al. 2019) and the role of environmental and physiological factors (Suttle 2022) suggest that outcomes could vary among different populations or conditions. So, more extended studies with larger sample sizes and direct assessment of mineral concentrations in tissues, such as the placenta and CL, are essential to verify the mechanisms.

In the present study, statistically, Hb concentration tended to be higher in the OTM group as compared with other groups. Other studies reported no significant impact of OTM supplementations on Hb of lambs, ewes, calves and pigs (Eckert et al. 1999; Creech et al. 2004; Dezfoulian et al. 2012; Alimohamady et al. 2019; Seifalinasab et al. 2022). However, other studies in different species indicated an improving effect of OTM supplementation on Hb concentration (Aksu and Ozsoy 2010; Sobhanirad and Naserian 2012; Qiu et al. 2022). Hb, as an iron‐containing protein transports oxygen in red blood cells, and iron supplementation increases Hb concentration, which is necessary during pregnancy with a high risk of anaemia (Santosa et al. 2022). The increase in the value of Hb in ewes fed OTM may be related to the higher bioavailability of iron in this group, which can be very supportive during pregnancy. The research faces a number of limitations. With only 40 ewes divided into four groups, the small sample size may reduce the statistical power and overlook minor effects. The brief five‐week period, focused around mating, does not allow for the evaluation of long‐term outcomes such as lamb development, ongoing reproductive success or the cumulative health impacts over different seasons.

## 5 Conclusions

Overall, supplementing the flushing diet with OTM based on advanced chelate technology enhanced the reproductive performance of ewes.

## Author Contributions

**Sedigheh Vatankhah**: methodology, project administration, writing – original draft. **Marziyeh Ebrahimi**: methodology, data curation, formal analysis, writing – review and editing. **Gholamali Moghaddam**: methodology. **Davoud Kianifard**: methodology. **Akbar Taghizadeh**: methodology. **Reza Asadpour**: methodology. **Mohammad Hassan Nazaran**: resources.

## Ethics Statement

The authors confirm that the ethical policies of the journal, as noted on the journal's author guidelines page, have been adhered to and the appropriate ethical review committee approval has been received. The authors confirm that they have followed EU standards for the protection of animals used for scientific purposes. As part of this experiment, all animal procedures and ethical considerations were performed following the Guide to the Care and Use of Agricultural Animals in Research and Teaching. Also, this study was conducted according to the procedures established by the Iranian Ministry of Agriculture (Experimental Authorization No. ASRI‐2016‐95014). FASS (2010) Guide for the care and use of agricultural animals in agricultural research and teaching, third ed. Consortium for developing a guide for the care and use of agricultural animals in agricultural research and teaching, Champaign, IL, USA.

## Conflicts of Interest

The authors declare no conflicts of interest.

## Data Availability

The data generated from this research study will be accessible by request through direct correspondence with the authors.
